# A proteomic analysis of the regulon of the NarP two-component regulatory system response regulator in the bovine pathogen *Mannheimia haemolytica *A1

**DOI:** 10.1186/1756-0500-4-510

**Published:** 2011-11-24

**Authors:** Ichiro Inamoto, Reggie Lo

**Affiliations:** 1 Department of Molecular and Cellular Biology, University of Guelph, Guelph, ON, N1G 2W1, Canada

**Keywords:** *narP *mutant, 2D electrophoresis, LC-MS

## Abstract

**Background:**

The response of the NarQP two-component signal transduction system regulon in response to the presence of nitrate for the bovine pathogen *Mannheimia haemolytica *A1 was investigated by proteomic analysis. Total proteins from a *narP *mutant and the parent SH1217 grown with or without NaNO_3 _supplement were examined by ISO-DALT 2D electrophoresis and liquid chromatography-mass spectrometry.

**Results:**

Seventeen proteins were differentially expressed in the parent strain SH1217 in response to the addition of NaNO_3 _to the growth media. These responses were absent in the *narP *mutant, indicating that the altered production of these proteins is mediated by NarP_*Mh*_. Interestingly, NarP_*Mh *_mediated the increased production of some proteins which are not generally associated with nitrate respiration, such as the iron transporters FbpA and YfeA. The increased production of proteins such as superoxide dismutase, SodA, and GAPDH were also observed. The increased production of these iron-regulated proteins by NarP_*Mh *_is thought to enhance the swift establishment of the nitrate respiration mechanism of *M. haemolytica *during pathogenesis.

**Conclusion:**

The data suggested NarP_*Mh *_acts as an important regulator which regulates the expression of a small set of proteins in response to nitrate availability. This may contribute to the prevalence of *M. haemolytica *A1 in its host during pathogenesis of BPP, through enhancing the effectiveness of nitrate respiration either directly or indirectly.

## Background

*Mannheimia haemolytica *A1 is a Gram-negative, non-motile coccobacillus normally found in the upper respiratory tract of healthy calves. This bacterium is an opportunistic pathogen which causes bovine pneumonic pasteurellosis (BPP), an acute pneumonia that often leads to death in the animals [1,2]. BPP usually occurs after transportation of calves from the farm to feedlot and is also known as 'shipping fever'. It has been estimated that over $1 billion is lost annually in North America due to this disease [3,4].

Environmental stresses such as transportation, crowding and viral infection also play a major role in the pathogenesis of BPP [5]. Exposure to these stress factors compromises the immune system of the animal allowing *M. haemolytica *A1 to multiply and infect the lung through aerosol and gravitational movement. Many virulence factors such as the leukotoxin, *O*-sialoglycoprotease and adhesins are expressed by the bacterium during infection which contribute to the disease [6,7]. Since *M. haemolytica *A1 is an opportunistic pathogen, expression of these virulence factors are likely to respond to specific environmental signal(s).

Two-component signal transduction systems (TCSs) are environmental response mechanisms commonly found in bacterial species and in some eukaryotes [8]. Previously, we have identified five putative pairs of TCSs in the *M. haemolytica *A1 genome; ArcBA, CpxRA, NarQP, PhoRB and TtrSR. Out of these, the nitrate sensory system, NarQP_*Mh*_, was further investigated by the generation of a *narP *knock-out mutant [9]. We showed that the production of some proteins was differentially regulated in the parental strain in response to the addition of nitrate to the media, while this response was absent in the *narP *mutant. Interestingly, the primary virulence factor, leukotoxin, and the periplasmic component of an iron ABC transporter, FbpA, showed increased production in response to additional nitrate in a NarP dependent manner. In this study, a proteomic approach using ISO-DALT 2D electrophoresis in combination with liquid chromatography-mass spectrometry (LC-MS) was used to identify the proteins regulated by the presence of nitrate in a NarP dependent manner.

## Results

### The *M. haemolytica *A1 proteome

Total proteins extracted from SH1217 grown without NaNO_3 _supplementation were applied to a 17 cm IEF strip (pH 5-8) and separated by isoelectric focusing. This pH range was chosen since no proteins were observed between pH 3-5 and pH 8-10 in a pilot study using a 7 cm IEF strip (pH 3-10) (results not shown). The proteins were separated in the second dimension by SDS-PAGE and visualized by Colloidal Coomassie blue staining.

**Figure 1:**
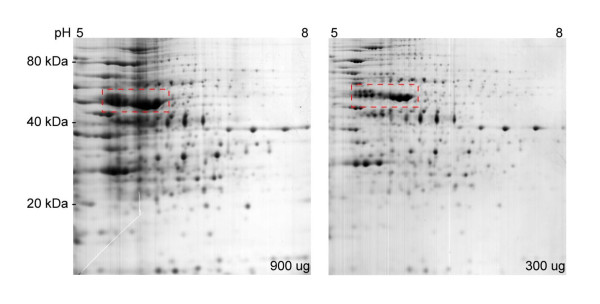
**The proteome of *M. haemolytica *SH1217 shown by ISO-DALT 2D electrophoresis**. A total protein load of 900 μg or 300 μg preparations from SH1217 grown with NaNO_3 _supplementation were separated by isoelectric focusing on a 17 cm IEF strip. The proteins were separated in the second dimension by SDS-PAGE and visualized by Colloidal Coomassie blue staining. *Dashed boxes *note some of the proteins expressed in greater abundance compared to the rest of the proteins.

The *M. haemolytica *A1 proteome contained a small number of proteins which were produced in much greater abundance compared to the rest of the proteins (Figure 1). These proteins are estimated to consist up to 15-20% of the total protein volume. Since a small number of proteins represent a large portion of the total protein concentration, loading the IEF strip with a conventional (lower) amount of total proteins causes an under-representation of the rest of the proteins. Thus, in subsequent studies, total protein samples were analyzed under two different protein loads: 300 μg of protein load to analyze the abundant proteins and 900 μg of protein load to detect the less abundant proteins.

### Differential production of proteins in *M. haemolytica *A1 in response to NaNO_3_

Either 900 or 300 μg of total proteins extracted from *M. haemolytica *A1 grown with or without NaNO_3 _supplementation were separated by large format ISO-DALT 2D electrophoresis using a 17 cm IEF strip (pH 5-8). The Colloidal Coomassie stained gels were scanned and analyzed using the BioNumerics Software looking for differentially expressed spots. Prior to this analysis, the volumes of the spots were normalized to the reference spot, shown to be present at a consistent level within the strains and treatments (spot 'N'; peptidylprolyl isomerase), to adjust for variability in protein loading between each gel.

**Figure 2:**
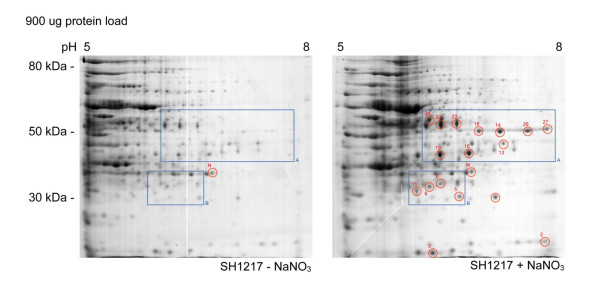
**Differential production of proteins in *M. haemolytica *SH1217 in response to NaNO_3 _supplementation in the media, shown by ISO-DALT 2D electrophoresis**. A 900 μg of total protein preparations from SH1217 grown with or without NaNO_3 _supplementation were separated by isoelectric focusing on a 17 cm IEF strip. The proteins were separated in the second dimension by SDS-PAGE and visualized by Colloidal Coomassie blue staining. *Red circles *note the spots which showed over 1.5 fold increase in their volume compared to the volume of the same spot in the other gel. These spots were identified by mass spectrometry, and are numbered according to Table 1. The spot used for normalization in statistical analysis is labeled 'N'.

**Figure 3:**
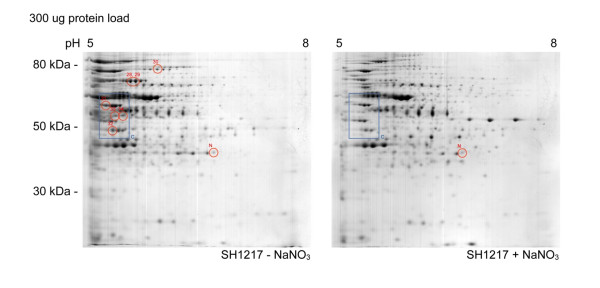
**Differential production of proteins in *M. haemolytica *SH1217 in response to NaNO_3 _supplementation in the media, shown by ISO-DALT 2D electrophoresis**. A 300 μg of total protein preparations from SH1217 grown with or without NaNO_3 _supplementation were separated by isoelectric focusing on a 17 cm IEF strip. The proteins were separated in the second dimension by SDS-PAGE and visualized by Colloidal Coomassie blue staining. *Red circles *note the spots which showed over 1.5 fold increase in their volume compared to the volume of the same spot in the other gel. These spots were identified by mass spectrometry, and are numbered according to Table 1. The spot used for normalization in statistical analysis is labeled 'N'.

**Figure 4:**
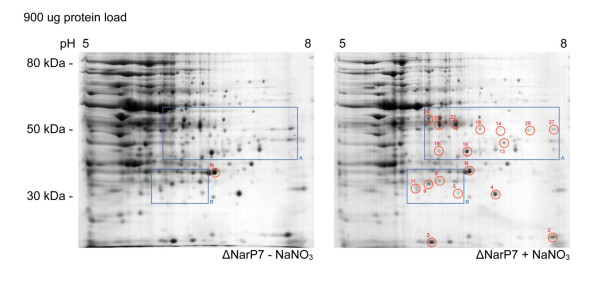
**Differential production of proteins in *M. haemolytica *ΔNarP7 in response to NaNO_3 _supplementation in the media, shown by ISO-DALT 2D electrophoresis**. A 900 μg of total protein preparations from SH1217 grown with or without NaNO_3 _supplementation were separated by isoelectric focusing on a 17 cm IEF strip. The proteins were separated in the second dimension by SDS-PAGE and visualized by Colloidal Coomassie blue staining. None of these spots were shown to be differentially produced in ΔNarP7 under the two NaNO_3 _conditions. The spot used for normalization in statistical analysis is labeled 'N'.

Twenty-seven spots were identified in SH1217 which were potentially differentially produced in response to NaNO_3 _supplementation with 900 μg of protein load (Figure 2). Seven additional spots were identified to be differentially produced in SH1217 with 300 μg of protein load, in response to NaNO_3 _supplementation (Figure 3). These responses were not observed when the same analyses were carried out on ΔNarP7 (Figure 4). The analysis was based on three biologically independent samples, each examined on three different gels (i.e. a total of 9 gels), and were shown to be statistically significant at *p *< 0.05.

### Identification of the differentially produced proteins by mass spectrometry

Out of the 34 spots identified above, 27 clearly separate ones were identified by liquid chromatography-mass spectrometry (Table 1). In some cases, a single protein was represented by multiple spots on the gel. A total of 20 different proteins were identified through mass spectrometry.

**Table 1:** Proteins differentially produced in response to NaNO_3 _supplementation determined by the large-format ISO-DALT 2D electrophoresis

Spot #	Differential production in SH1217^a^	Differential production in ΔNarP7	Identification (accession number)	MS Coverage (%)
2	2.17	no response	50S ribosomal protein RplI (197748946)	52

3	1.82	no response	Ribosomal protein S6 (53729064)	53

4/5	1.88/1.69	no response	Peptide chain release factor RF4/ribosome recycling factor (RRF) (197750015)	51/26

8	2.47	no response	Manganese dependent superoxide dismutase (51292145)	25

9	1.72	no response	Xanthine-guanine phosphorybosyltransferase (113460152)	15

11	1.64	no response	3-oxoacyl-[ACP]-reductase (197747648)	38

13	2.19	no response	Hypothetical protein (OmpH?) (197749776)	8

14/18/26/27	4.09/3.7/only in SH+/only in SH+	no response	Iron (Fe^3+^) ABC Transporter, FbpA (197750010)	37/31/29/30

16/19	2.66/3.13	no response	Iron Transporter, YfeA (110735183)	38/29

22/23	2.75/2.93	no response	Glyceraldehyde-3-phosphate dehydrogenase (197749950)	29/29

24	3.29	no response	Aspartate transaminase (197749486)	24

28/29	0.27/0.34	no response	Phosphophenolpyruvate carboxykinase (197749783)	30/21

30	0.34	no response	2',3'-cyclic-nucleotide 2'-phosphodiesterase (197747925)	13

31	0.32	no response	Phosphoglycerate kinase (197750198)	55

32	Only in SH -	no response	Elongation factor EF1B (197750012)	36

33	Only in SH -	no response	PTS family mannose porter component IIAB (197748528)	9

34	Only in SH -	no response	L-asparaginase II (197747589)	19

^a^fold-change

**Figure 5:**
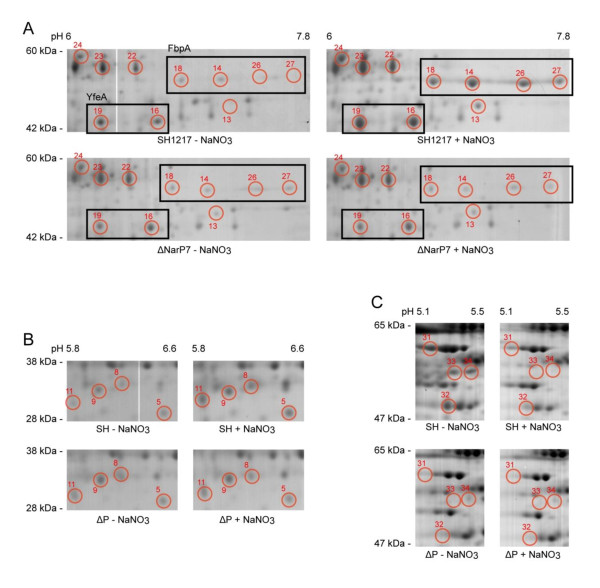
**Over-production of FbpA and YfeA in response to the addition of NaNO_3 _supplementation in SH1217**. Segments of large-format ISO-DALT 2D for SH1217 and ΔNarP7 grown with or without NaNO_3 _are shown. All gels were loaded with 900 μg of total protein. The *boxes *indicate the spots representing FbpA and YfeA.

The spots were identified by liquid chromatography-mass spectrometry. The spot number corresponds to Figures 2, 3, 4 and 5. Some spots represented the same protein. Differential production of the spots is shown by the following ratio: [volume of the spot when grown with additional NaNO_3_/volume of the spot when grown without additional NaNO_3_]. Some spots were produced only when SH1217 was grown with NaNO_3 _supplementation (Only in SH+) or without NaNO_3 _supplementation (Only in SH-). The change in the volume of these spots in response to NaNO_3 _supplementation was not observed in ΔNarP7 (no response)

The differential production of 17 of the 20 proteins were verified as statistically significant (two-tailed, unpaired t-test (*p *< 0.05)). For these proteins, 11 and 6 proteins showed an increased or decreased production, respectively. About half of these proteins were involved in metabolic processes, while other proteins were involved in processes such as transcription and translation. Two iron transport proteins were found on this list; FbpA and YfeA (Figure 5), both of which were strongly over-produced in response to additional NaNO_3_. None of these proteins were differentially produced in ΔNarP7 suggesting that the production of these proteins are under direct or indirect control of NarP.

## Discussion

The present study is one of the few reports of the NarP regulon in Pasteurellaceae, along with the study by Ravcheev *et al*. which was based entirely on *in silico *methods [10]. Using a proteomic approach, we identified 17 proteins which were differentially produced in response to NaNO_3 _supplementation in SH1217. This response is NarP_*Mh *_dependent since no alteration in the protein levels were observed in the isogenic *narP *mutant.

The production of two iron transport proteins was found to be regulated by NarP_*Mh*_. FbpA is the periplasmic component of an iron ABC-transport system, which was produced at over four-fold higher levels in SH1217 in response to NaNO_3 _supplementation. This agrees with our previous observation [9]. Another iron transporter, YfeA, was expressed at least 2.6 fold higher in response to NaNO_3 _supplementation. YfeA is a periplasmic iron-binding protein of the YfeABCD iron ATP-transporter system, which transports chelated iron across the inner membrane [11]. This system was first identified in *Yersinia pestis *and is required for the full virulence of the pathogen [12]. Other HAP organisms such as *H. influenzae, A. pleuropneumoniae *and *P. multocida *carry *yfeABCD *homologues, but all are regulated by iron [13-16]. The NarP mediated nitrate control of YfeA expression has not been reported previously.

Both FbpA and YfeA were represented by multiple, horizontally aligned spots on the 2D gel, having similar masses but different pIs. The separate spots likely represent different isoforms of the proteins generated by post-translational modifications such as phosphorylation or removal of a charged residue, which alter the pI without significantly changing its mass. For instance, FbpA has been shown to be truncated into a slightly smaller 35.5 kDa form after translation [17]. Further, *M. haemolytica *A1 FbpA binds to ferric iron using a synergistic carbonate anion to stabilize the ligand [18], and therefore the four spots for FbpA may represent the protein bound to its ligands in different combinations (FbpA alone, FbpA + Fe^3+^, FbpA + carbonate, FbpA + Fe^3+ ^+ carbonate). An observation that transferrin, which shares structural/functional similarity to FbpA, remains bound to its ligands during urea-PAGE also support this possibility [19].

Interestingly, expression of *napA *and its related proteins are heavily dependent on iron, as many of them require the Fe_4_-S_4 _cluster for their function [20]. Studies have shown a down-regulation of the *nap *operon by the ferric regulator Fur in response to iron deprivation in *M. haemolytica, A. pleuropneumoniae, P. multocida*, and *E. coli *[13-15,21], which frees iron for proteins involved in more crucial functions. Thus, we hypothesize that this operon is down-regulated when the microorganism multiplies in the bovine lung where iron is scarce. When it is necessary to carry out nitrate respiration, NarQP_*Mh *_system overcomes this negative regulation on NapA by up-regulating several iron transporters such as FbpA and YfeA. The increased iron availability brought by these proteins counteracts the down-regulating effects on the *nap *promoter from Fur.

Production of superoxide dismutase (SodA) was 2.17 fold higher in response to NaNO_3 _supplementation. Two forms of superoxide dismutases exist in *E. coli*: the manganese dependent SodA and the iron dependent SodB [22], *M. haemolytica *A1 only possess SodA. These enzymes protect the cellular components from the reactive oxygen intermediates generated through oxygen metabolism [23]. The importance of SodA in *M. haemolytica *A1 virulence has been implicated as they may protect the bacteria from the oxidative damage caused by neutrophils under leukotoxin attack [24]. The up-regulation of *sodA *by the NarP system has been reported in *E. coli *[25]. Interestingly, Raveheev *et al*. did not identify *sodA *as part of the NarP regulatory network [10]. It is possible that NarP regulates *sodA *indirectly.

The up-regulation of *napA *by the NarQP system in response to environmental nitrate is well established in other organisms [26,27]. In contrast, the over-production of NapA was not detected in our analysis in response to NaNO_3_. However, a perfect NarP binding sequence was identified in the promoter region of the *M. haemolytica *A1 *nap *operon (data not shown) which still supports the idea that these genes are regulated by NarP_*Mh*_.

In many microorganisms, the major role of NarQP is to regulate the expression of nitrate respiration systems, presumably providing an alternate respiration pathway in the oxygen deprived lungs during BPP pathogenesis. This, of course, is mainly accomplished through the production of proteins in the respiratory chain. However, our data suggest that NarQP_*Mh *_also regulates the expression of certain proteins such as FbpA and YfeA which does not directly take part in nitrate respiration, but instead aid in the efficient establishment of the system. Further, the system may also play an important role in controlling the production of factors important for virulence, such as SodA (this study) and leukotoxin [9].

This is the first report of the role of NarP_*Mh *_regulon in *M. haemolytica *A1, showing that it has expanded its control over the expression of proteins otherwise not known to be controlled by the system in other organisms. This may be a shared trait in Pasteurellaceae, as similar observations were made by Ravcheev *et al*. [10] for other members of the family using an *in silico *method based on nucleotide sequence recognition profiles for NarP binding sites. Interestingly, very few genes/proteins were commonly found between our approach and the *in silico *method. A combination of two reasons explains this difference: 1) we were able to detect proteins in the NarP regulon which were missed by the *in silico *method; and 2) the result simply represent the difference between the NarQP systems in *M. haemolytica *against other members of its family. Nonetheless, our results provide experimental evidence suggesting the expanded role NarQP system in Pasteurellaceae.

## Material and methods

### Bacterial strains and growth conditions

*M. haemolytica *A1 strain SH1217 was obtained from Dr. Sarah Highlander, Baylor College of Medicine. The *M. haemolytica *A1 *narP *knock-out mutant strain, ΔNarP7, was generated from SH1217 [9]. *M. haemolytica *A1 was cultured in brain heart infusion broth (BHIB) at 37°C. To examine the response to the addition of nitrate, BHIB was supplemented with 2.5 mM NaNO_3_. *M. haemolytica *A1 was grown in a semi-anaerobic condition by growing the liquid cultures in a sealed container without shaking modified from [28].

### Extraction of *M. haemolytica *A1 proteins

An overnight culture of *M. haemolytica *A1 was subcultured in a 1/100 dilution into 750 mL of pre-warmed BHIB supplemented with 2.5 mM NaNO_3_. The culture was incubated semi-anaerobically at 37°C for 5-6 h. The cells were collected by centrifugation (7,818 × g), and the pellet was washed by resuspending in 30 mM Tris Base (pH 10.8). The cells were collected again by centrifugation (10,270 × g) and lysed in 10 mL of the 2D electrophoresis loading buffer (8 M Urea (Fisher) and 4% (w/v) CHAPS (Sigma-Aldrich) in 30 mM Tris Base pH 10.8) on ice for 5 min. Cell debris were removed by centrifugation at 17590 × g. The extracted protein sample was washed by acetone precipitation (4 volumes of acetone to a volume of the sample), and resuspended in the 2D electrophoresis loading buffer.

The protein concentration of this sample was quantified using the Quick Start™ Bradford Assay kit (Bio-Rad). The OD_595 _of the samples were determined using a spectrophotometer (DU^®^730), and their concentrations were calculated using a standard curve generated from serial dilutions of bovine serum albumin, using the onboard program of the spectrophotometer.

Protein samples from two strains, each grown in two different conditions were extracted by this method: SH1217 grown with and without NaNO_3 _supplementation; and ΔNarP7 grown with and without NaNO_3 _supplementation.

### Sample preparation and ISO-DALT 2D electrophoresis

ISO-DALT 2D electrophoresis was performed using the large format, 17 cm IEF strip (Bio-Rad). The protein samples were diluted appropriately in the rehydration solution (8 M Urea, 4% (w/v) CHAPS, 10 mM DTT (Bio-Rad) and 0.2% (v/v) Bio-Lyte^® ^3/10 Ampholyte (Bio-Rad). The diluted protein sample was applied to the IEF strip, and the strip was left to rehydrate at RT for 12-16 h. Iso-electric focusing was carried out in the Protean IEF Cell (Bio-Rad), with the following parameters: removal of small ions at 250 V for 1 h; removal of small ions at 500 V for 2 h; rapid voltage ramping to 10,000 V for 3 h; iso-electric focusing at 10,000 V for a total of 60,000 V-hours. Once iso-electric focusing was complete, the IEF strips were equilibrated with the equilibration buffer (6 M Urea, 2% (w/v) SDS, 20% (v/v) Glycerol, 130 mM DTT with a trace amount of bromophenol blue (Fisher) in 0.375 M Tris-HCl, (pH 8.8) for 15 min. The strip was equilibrated for a second time for 15 min in the equilibration buffer which contained 135 mM iodoacetamide (Sigma-Aldrich) in place of DTT. Finally, protein on the equilibrated IEF strips were electrophoresed in the second dimension on a large format SDS-PAGE gel. The gels were visualized by Colloidal Coomassie blue staining shown in [29].

### Quantitative analysis

The stained gels were scanned using the GS-800 Calibrated Densitometer (Bio-Rad Laboratories) at the highest resolution. These images were imported to and analyzed by the BioNumerics software (v5.1, Applied Maths). Briefly, protein spots were detected and quantified on each image. The volumes of these spots were then normalized using the reference spot 'N' (peptidylprolyl isomerase) in each gel to allow the volumes of each spot to be compared between different gels. Queries using Boolean operators were used to detect protein spots which were over-expressed or under-expressed in the protein samples of SH1217 grown with NaNO_3 _supplementation, as compared to the protein samples of SH1217 grown without the supplementation. A similar analysis was done to determine the differential protein expression in ΔNarP7 in response to NaNO_3 _supplementation. These analyses were repeated using three biologically independent samples, and the statistical significance of the differential protein expressions were assessed in a two-tailed, unpaired t-test (*p *< 0.05). A figure showing the gels from 2 biological samples of SH1217 grown without NaNO_3 _supplementation at a) 900 and b) 300 μg loading demonstrating reproducibility of the gels (Additional file 1: Figure S1).

### Mass Spectrometry analysis of differentially expressed proteins

The spots representing the differentially expressed proteins were manually cut out from the Colloidal Coomassie-stained gel. The gel fragments were subjected to in-gel trypsin digestion and analyzed by LC-MS at the Mass Spectrometry Facility, University of Guelph. Peptide mass spectra were obtained with a Bruker Reflex IV mass spectrometer. The data was used to search against the NCBI non-redundant protein sequence database using the MS Fit algorithm [30] for proteins matching the peptide mass spectra.

## Competing interests

The authors declare that they have no competing interests.

## Authors' contributions

II and RL designed the experimental approach. II carried out most of the laboratory work. Both II and RL analyzed the data and wrote the manuscript.

## Supplementary Material

Additional file 1 **Figure S1**. Click here for file
