# Supplementary figures and images for: A proteomic analysis of the regulon of the NarP two-component regulatory system response regulator in the bovine pathogen Mannheimia haemolytica A1
Source: BMC Res Notes. 2011 Nov 24;4:510. doi: 10.1186/1756-0500-4-510 (PMC3262028; doi:10.1186/1756-0500-4-510)

a)
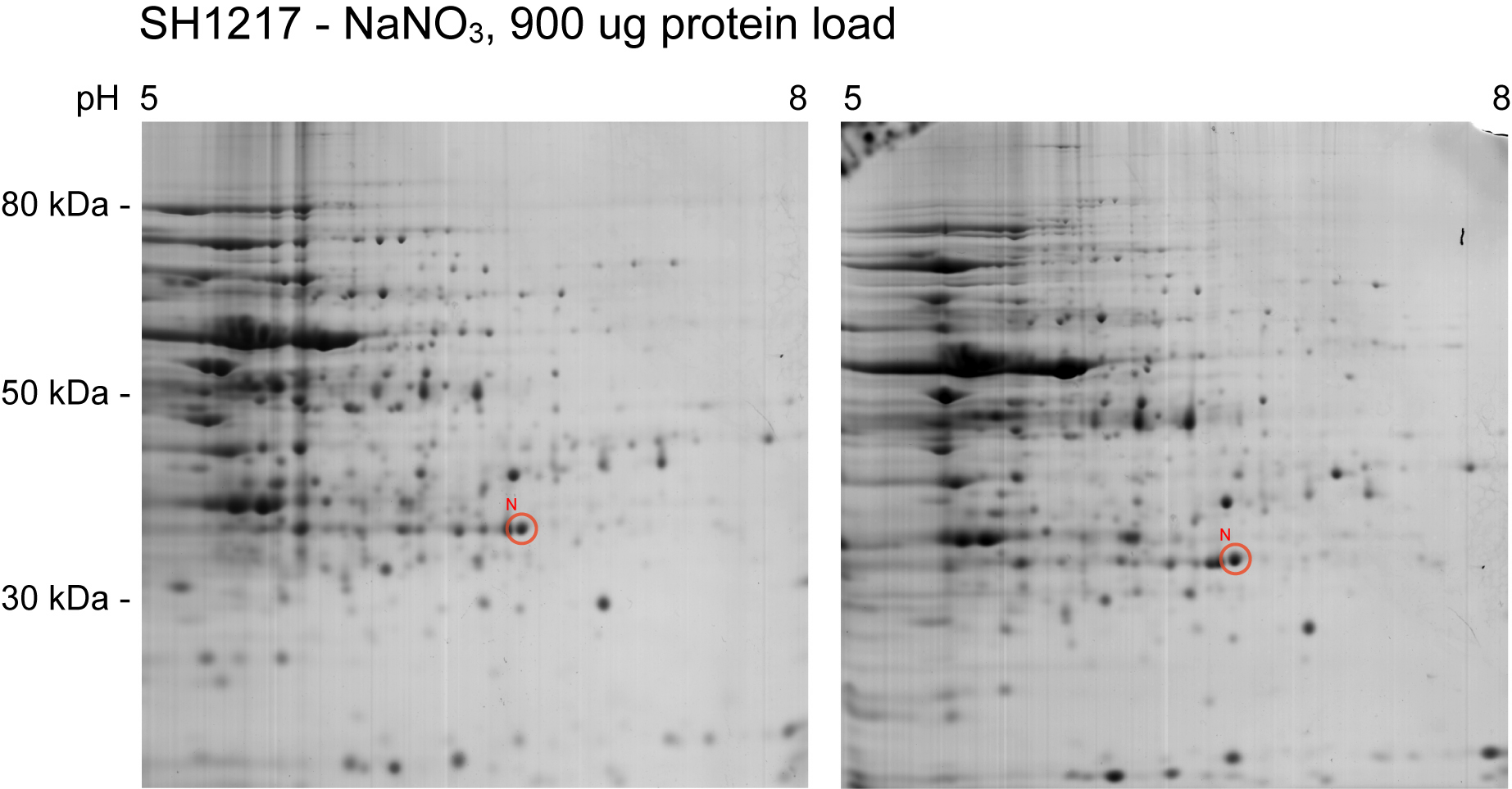


b)
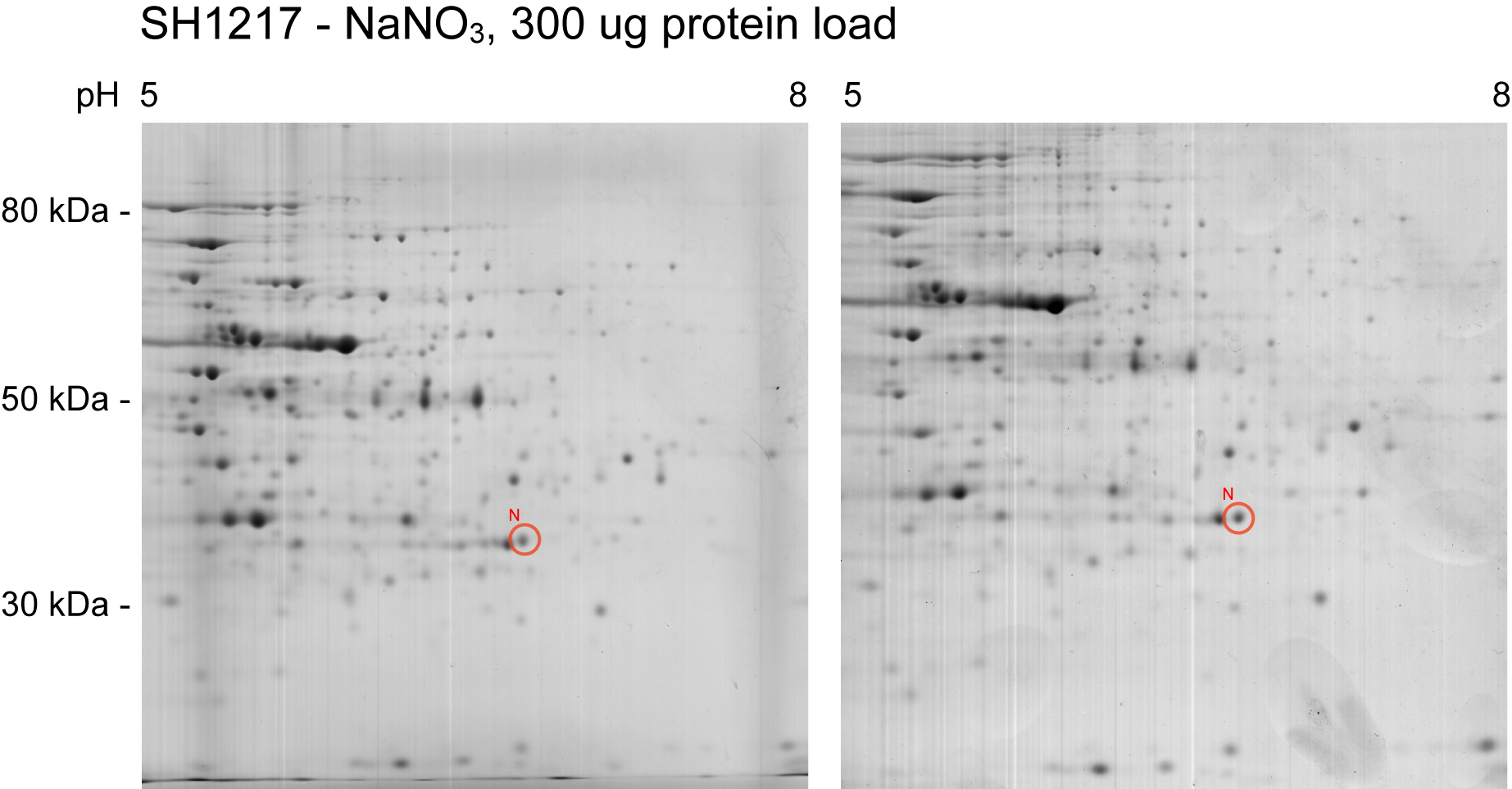


Supplementary Figure 1.

Supplement: Additional file 1 — Figure S1. [file 1756-0500-4-510-S1.DOC]
